# Evaluation of effectiveness and safety of amlodipine/valsartan/hydrochlorothiazide single-pill combination therapy in hypertensive patients: an observational study

**DOI:** 10.3109/21556660.2014.884506

**Published:** 2014-01-16

**Authors:** Andreas Hagendorff, Ira Kurz, Alfons Müller, Sven Klebs

**Affiliations:** 1 Universitätsklinikum Leipzig, Innere Medizin, Fachbereich Kardiologie/Angiologie, LeipzigGermany; 2 Clinical Research, Kantar Health GmbH, MünchenGermany; 3 Clinical and Regulatory Affairs, Novartis Pharma GmbH, NürnbergGermany

**Keywords:** Hypertension, Amlodipine, Combination, Hydrochlorothiazide, Observational study, Triple therapy, Valsartan

## Abstract

**Objective:**

This study evaluated the effectiveness and safety of amlodipine/valsartan/hydrochlorothiazide (A + V + H) single-pill combination therapy in the treatment of hypertensive patients in daily practice.

**Design and methods:**

This prospective, open-label, observational study, enroled adults for whom their physician considered treatment with the single pill combination as indicated. The observational period per patient was ∼3 months. Results were evaluated using basic descriptive statistical methods.

**Main outcome:**

Data of 7132 patients were analyzed. At baseline, the mean blood pressure (BP) was 158.8 ± 17.7 mmHg (systolic, sBP) and 91.5 ± 10.7 mmHg (diastolic, dBP). The most common cardiovascular risk factors were positive family history, dyslipidemia, and diabetes mellitus. The most commonly used daily doses of A + V + H at study end were 5/160/12.5 mg (30.5%) or 10/160/12.5 mg (33.1%). At the last visit mean BP was 135.0 ± 11.8 mmHg (sBP) and 80.2 ± 7.3 mmHg (dBP). The mean BP reduction at last visit compared with baseline was −23.7 ± 17.5 mmHg (sBP) and −11.3 ± 10.6 mmHg (dBP); 43.5% of the patients reached normalization (BP <140/90 mmHg for non-diabetics or <130/80 mmHg for diabetics) and 71.3% reached therapeutic response (sBP <140 or ≥20 mmHg decrease vs baseline and dBP <90 or ≥10 mmHg decrease vs baseline in non-diabetic patients and sBP <130 mmHg or ≥20 mmHg decrease vs baseline and dBP <80 mmHg or ≥10 mmHg decrease vs baseline in patients with diabetes). Adverse events (AEs) were recorded in 2.3% of the patients, the most frequent being peripheral edema (0.6%) and dizziness (0.2%).

**Conclusions:**

In daily practice, A + V + H single-pill treatment effectively lowered the average BP in patients with essential hypertension and was well tolerated.

## Introduction

Hypertension is the main cause for cardiovascular (CV) diseases^1^. Additionally, hypertension is among the leading causes of death worldwide. In 2001, 13.5% of the global total deaths were caused by hypertension-related diseases^2^. The complications and comorbidities associated with hypertensions include cerebrovascular disease, ischemic heart disease, cardiac and renal failure and stroke, as well as retino- and nephropathy^3,4^. In the last decades the prevalence of hypertension has increased and it is expected to do so further^5,6^. By 2008 the prevalence was ∼30% and for about half of the hypertensive patients blood pressure (BP) was under control (systolic BP [sBP] of <140 mmHg and diastolic BP [dBP] of <90 mmHg)^5^. Increasing longevity, obesity, physical inactivity, and an unhealthy diet are among the reasons for the increasing prevalence of hypertension^3^.

Several guidelines address the management of hypertension^7–9^. According to the European Society for Hypertension (ESH)/European Society of Cardiology (ESC)^8^ guidelines for most patients, including those with marked BP elevation, high CV risk or those needing to achieve a lower BP target, a combination of ≥2 drugs from different classes is advised to reach the target BP of <140/90 mmHg (<130/80 mmHg for diabetic and hypertensive high-risk patients). The specific BP target for diabetics has been re-appraised in the ESC/ESC hypertension guidelines and is now <140/90 mmHg also^9^.

Compliance is significantly influenced by the number of pills a patient is prescribed to take^10–12^. This ‘pill burden’ is further intensified by the high rate of comorbidities that often need treatment. Therefore, single-pill combination regimens have been developed. In addition to increased adherence, combination therapies can have beneficial effects on hypertension control when drugs with different mechanisms are combined^12,13^. For example, the combination of the calcium channel blocker amlodipine (A) and the angiotensin II receptor blocker valsartan (V) has shown to have an improved efficacy and safety profile compared with monotherapy^13–15^. When hydrochlorothiazide (H) was added to A + V the resulting single pill combination A + V + H lowered BP more effectively compared with the respective dual therapies^16^.

The aim of the present non-interventional study was to investigate the effectiveness of single-pill A + V + H therapy in daily practice and to evaluate the incidence and profile of occurring adverse events. Effectiveness of A + V + H therapy was evaluated with regard to normalization of BP according to the Guidelines of the ESH^8^ (<140/90 mmHg for non-diabetics or <130/80 mmHg for diabetics—as defined in the guidelines at the time of conducting the study) and therapeutic response (sBP <140 or ≥20 mmHg decrease vs baseline and dBP <90 or ≥10 mmHg decrease vs baseline). Additionally, the influence of the pill burden both on hypertensive patients and their treating physicians was investigated. These results are the subject of a separate publication^17^.

## Patients and methods

### Study design

This was a prospective, open-label, observational multi-center study conducted between November 2009 and November 2010. A total of 1269 practices of general practitioners and internists in Germany participated. The study was notified in accordance with §67 (6) German Drugs Law (AMG) and conducted according to the applicable regulatory requirements and recommendations. All procedures followed were in accordance with the ethical standards of the responsible committee on human experimentation (institutional and national) and with the Helsinki Declaration of 1975, as revised in 2000, and as far as applicable for observational, non-interventional studies. Informed consent was obtained from all patients for being included in the study. The participating physicians received a compensation for the documentation of each patient in accordance with the official scale of physicians’ fees (Gebührenordnung für Ärzte, GOÄ). The study was approved by the respective Ethics Committee.

The study has been registered according to local regulations in a German register: http://www.vfa.de/de/arzneimittel-forschung/datenbanken-zu-arzneimitteln/nisdb/nis-details/_382.

Treatment was performed only according to medical and therapeutic needs. Procedures and decisions including frequency and timing of examinations were the responsibility of the treating physician and were to be performed according to practice routine. Additional examinations exceeding the usual scope were not required.

### Study population

Data of 7132 patients of both genders diagnosed with essential hypertension and for whom treatment with A + V + H was indicated according to the physician’s opinion were analyzed in this non-interventional study. There were no additional inclusion criteria (e.g., severity of hypertension). Except for the contraindications mentioned in the respective summary of product characteristics (SmPC), no other exclusion criteria were applied. All dosages of A + V + H available on the German market could be used.

### Study conduct and assessments

The observational period per patient was 3 ± 1.5 months. Examinations were to be documented by the physician at the start and end of the observation period (after ∼12 weeks). Optionally, an additional visit (after ∼6 weeks) within this period could be documented. At the start of the study demographic and diagnostic data, CV risk factors, prior and concomitant diseases relevant to the indication, information on potential antihypertensive pre-treatment, and total number of pills (overall and for the treatment of hypertension) were collected. Vital signs (sBP and dBP, heart rate) were assessed according to clinical practice and documented, as well as dosing of A + V + H therapy, dosing and intake of concomitant antihypertensive therapy, at study start, end, and in between visits. Data from laboratory parameters or ambulatory blood pressure measurements was not collected. Adverse events (AEs) were reported during the whole study period according to local regulations.

At the start of the observational period, patients filled in a questionnaire concerning the influence of the number of pills, and physicians filled in a questionnaire concerning the impact of the number of pills in daily practice. Results regarding pill burden are the subject of a separate publication^17^.

The data in all documentation forms was examined for their plausibility by the data management department. Additionally, on site monitoring and source data validation was carried out for a defined percentage (2%, in line with common practice in Germany^18^ of randomly chosen study centers).

### Data analysis

According to the pre-defined statistical analysis plan, statistical evaluation was performed using basic descriptive statistical methods and explorative interpretation of the results. The results of the overall population are shown. For some analyses, additional sub-groups (by sBP, dBP, and age at study start) are presented. The statistical evaluation was carried out using SAS Version 9.2 for Windows (SAS Institute, Cary, NC).

For qualitative variables, the absolute and relative frequencies were given and, for quantitative variables, characteristics of statistical distribution (e.g., Mean, Median, Minimum, Maximum, and Standard Deviations) were calculated. Missing data were not replaced for analysis and are not always in the tables or figures. Hence, the total across the respective categories does not always yield 100% for each of the parameters.

To minimize the influence of cases of premature discontinuations and those lost to follow-ups, for each patient the data of the last available visit after baseline (either the optional visit after ∼6 weeks or the visit at end of observation after ∼12 weeks) were used and summed up as a last follow-up (called ‘last visit’).

## Results

### Patient demographic characteristics

Baseline characteristics are summarized in Table 1. The percentage of male patients was slightly higher compared with female patients, and the mean age was 64.8 ± 11.5 years. The mean height was 170.6 ± 9.2 cm, with a mean weight of 85.9 ± 16.2 kg. The mean BMI was 29.5 ± 5.0 kg/m^2^. BMI categories showed that the majority of patients were overweight (45.5%) or obese (37.7%).

**Table 1.** Demographic and baseline parameters.

	*n* (%) or Mean ± SD	Median
Total	7132 (100.0)	
Gender		
Male	3695 (51.8)	
Female	3263 (45.8)	
Missing	174 (2.4)	
Age (years) at start of observation	64.8 ± 11.5	66.0
Age categorization
<65 years	3327 (46.6)	
≥65 and <80 years	3110 (43.6)	
≥80 years	658 (9.2)	
Missing	37 (0.5)	
Height (cm)	170.6 ± 9.2	170.0
Weight (kg)	85.9 ± 16.2	84.0
BMI (kg/m^2^)	29.5 ± 5.0	28.7
BMI categories
Normal body weight (<25 kg/m^2^)	1083 (15.2)	
Pre-adiposity (≥25 and <30 kg/m^2^)	3235 (45.4)	
Adiposity (≥30 kg/m^2^)	2689 (37.7)	
Missing	125 (1.8)	
Systolic blood pressure at study start	158.8 ± 17.7	160.0
Systolic blood pressure (categories) at study start
<140 mmHg	663 (9.3)	
≥140 and <160 mmHg	2715 (38.1)	
≥160 and <180 mmHg	2668 (37.4)	
≥180 mmHg	1071 (15.0)	
Missing	15 (0.2)	
Diastolic blood pressure at study start	91.5 ± 10.7	90.0
Diastolic blood pressure (categories) at study start
<80 mmHg	546 (7.7)	
≥80 and <90 mmHg	1755 (24.6)	
≥90 and <100 mmHg	2818 (39.5)	
≥100 and <110 mmHg	1586 (22.2)	
≥110 mmHg	414 (5.8)	
Missing	13 (0.2)	
Essential hypertension		
Yes	7106 (99.6)	
Missing	26 (0.4)	
Duration of essential hypertension (years)	8.9 ± 7.1	7.8
Duration of essential hypertension (years) categories
<1 year	637 (8.9)	
≥1 and <5 years	1796 (25.2)	
≥5 and <10 years	1855 (26.0)	
≥10 years	2446 (34.3)	
Missing	398 (5.6)	
Cardiovascular risk factors (most common)		
Positive family history	4328 (60.7)	
Dyslipidemia	3342 (46.9)	
Diabetes mellitus	2415 (33.9)	
Smoker	1865 (26.1)	
Coronary heart disease	1625 (22.8)	
Left ventricular hypertrophy	1583 (22.1)	
Missing	313 (4.4)	
Anti-hypertensive pre-treatment		
Anti-hypertensive pre-treatment	6659 (93.4)	
No anti-hypertensive pre-treatment	449 (6.3)	
Pre-treatment not specified	7 (0.1)	
Missing	17 (0.2)	
Prior anti-hypertensive medication (most common)		
Hydrochlorothiazide	3160 (47.5)	
Amlodipine	2663 (40.0)	
Ramipril	1484 (22.3)	
Bisoprolol	928 (13.9)	
Metoprolol	801 (12.0)	
Torasemide	696 (10.5)	

SD, standard deviation; kg, kilogram; m^2^, square-meter; mmHg, millimeters of mercury; cm, centimeter; BMI, body mass index.

Essential hypertension was diagnosed for 99.6% of patients. For 0.4% of patients the information was missing. At baseline the mean sBP was 158.8 ± 17.7 mmHg and the mean dBP was 91.5 ± 10.7 mmHg.

The most common risk factors were positive family history (60.7%), dyslipidemia (46.9%), and diabetes mellitus (33.9%). Before start of observation with A + V + H, 93.4% of the patients had received antihypertensive medication, with the most common compounds being H (47.5%) and A (40.0%).

### Treatment with A + V + H

For the majority of patients (98.7%) a visit at the end of the observation period after ∼12 weeks was documented. Additionally, 74.5% of patients had a documented visit during the observation period. The mean time between baseline and the first or second visit was 1.6 ± 1.2 (∼6 weeks) and 3.0 ± 1.5 months (∼12 weeks), respectively.

Table 2 presents the A + V + H dosages from the start of the study until the last observation. The most commonly reported daily doses of A + V + H at study end were 5/160/12.5 mg (30.5%) or 10/160/12.5 mg (33.1%).

**Table 2.** Treatment with single-pill combination of amlodipine/valsartan/hydrochlorothiazide (A + V + H), daily dose.

Daily dose of A + V + H (mg)	Start of observation		6 weeks		12 weeks		Last visit	
	*n*	%	*n*	%	*n*	%	*n*	%
Total	7132	100.0	5312	100.0	7040	100.0	7108	100.0
5/160/12.5 mg	2644	37.1	1617	30.4	2143	30.4	2165	30.5
10/160/12.5 mg	2251	31.6	1810	34.1	2334	33.2	2351	33.1
5/160/25 mg	547	7.7	349	6.6	494	7.0	498	7.0
10/160/25 mg	907	12.7	763	14.4	1038	14.7	1041	14.6
10/320/25 mg	772	10.8	675	12.7	961	13.7	971	13.7
Missing	11	0.2	98	1.8	70	1.0	82	1.2

mg, milligram; *n*, number.

The change in A + V + H dosage is presented in Table 3. For the majority of patients (>75%), the A + V + H dosage remained stable from baseline compared with the last visit. For 14.1% of the patients receiving 5/160/12.5 mg and 15.0% of patients receiving 5/160/25 mg A + V + H the dosage of A was increased from 5 to 10 mg. About 10% of patients receiving 10/160 mg/25 mg A + V + H increased V from 160 to 320 mg.

**Table 3.** Changes in treatment with amlodipine/valsartan/hydrochlorothiazide (A + V + H).

Start of observation	Total		Last visit											
			5/160/12.5 mg		10/160/12.5 mg		5/160/25 mg		10/160/25 mg		10/320/25 mg		Missing	
	*n*	%	*n*	%	*n*	%	*n*	%	*n*	%	*n*	%	*n*	%
Total	7108	100.0	2165	30.5	2351	33.1	498	7.0	1041	14.6	971	13.7	82	1.2
5/160/12.5 mg	2638	100.0	2057	78.0	372	14.1	49	1.9	62	2.4	63	2.4	35	1.3
10/160/12.5 mg	2242	100.0	69	3.1	1939	86.5	8	0.4	127	5.7	77	3.4	22	1.0
5/160/25 mg	547	100.0	15	2.7	7	1.3	415	75.9	82	15.0	24	4.4	4	0.7
10/160/25 mg	905	100.0	8	0.9	17	1.9	18	2.0	758	83.8	93	10.3	11	1.2
10/320/25 mg	769	100.0	13	1.7	16	2.1	7	0.9	11	1.4	714	92.8	8	1.0
Missing	7	100.0	3	42.9	0	0.0	1	14.3	1	14.3	0	0.0	2	28.6

mg, milligram; *n*, number.

### BP response

**Figure 1.**
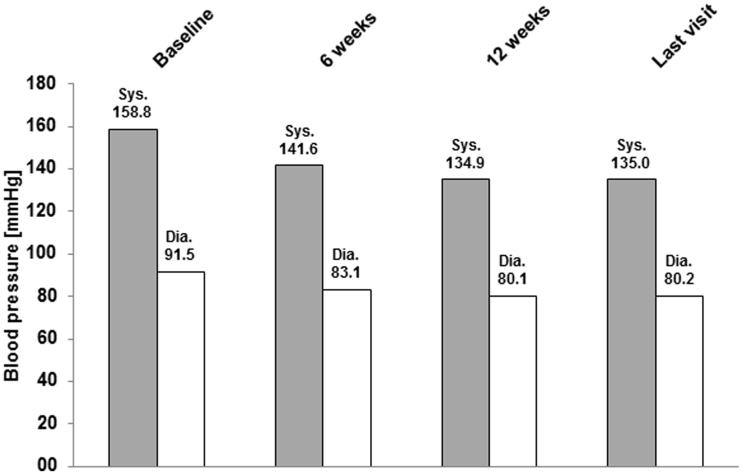
Time course of systolic and diastolic blood pressure—Mean values. mmHg, millimeters of mercury; sys, systolic; dia, diastolic.

During the course of the observation period, BP decreased markedly (Figure 1). From the start of the observation to the end of the observation, the mean sBP decreased from 158.8 ± 17.7 to 135.0 ± 11.8 mmHg and the mean dBP decreased from 91.5 ± 10.7 to 80.2 ± 7.3 mmHg. The mean BP difference at the last visit compared with baseline was −23.7 ± 17.5 mmHg (sBP) and −11.3 ± 10.6 mmHg (dBP).

**Figure 2.**
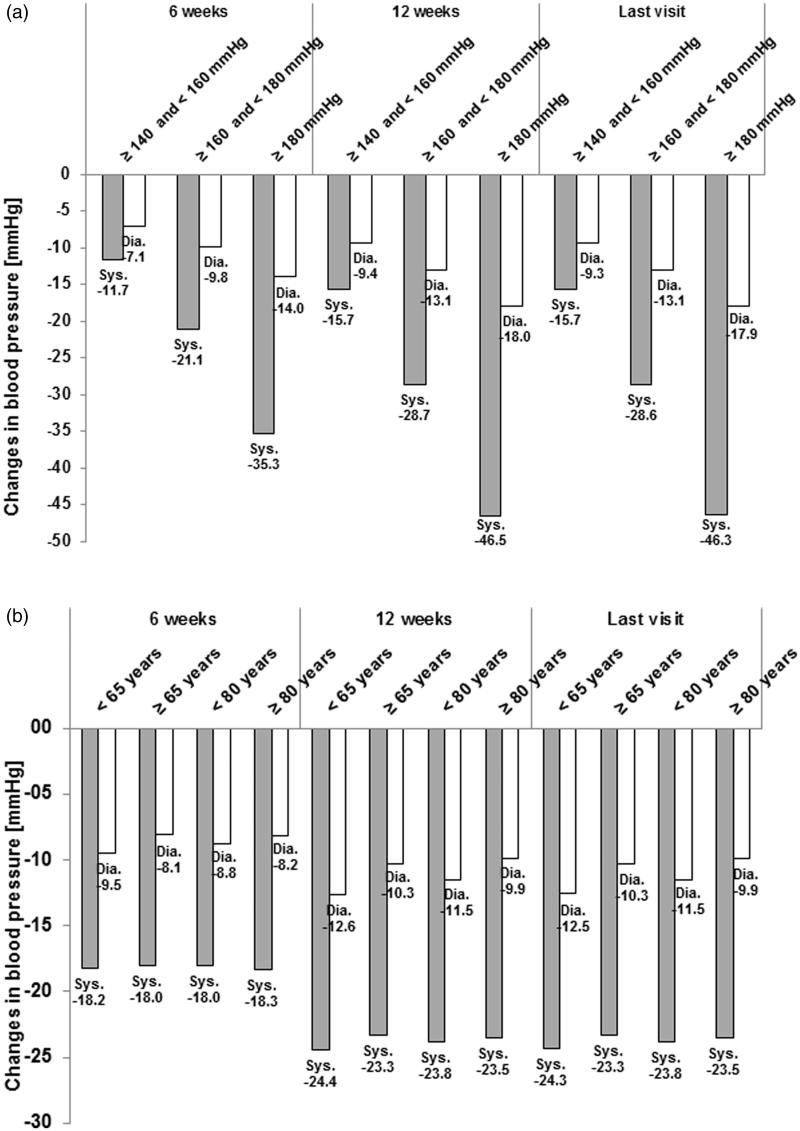
Absolute changes in blood pressure from start of observation. (a) Stratified by sBP at start of observation—Mean values. (b) Stratified by age at start of observation—Mean values. mmHg, millimeters of mercury; sys, systolic; dia, diastolic.

The degree of BP reduction in relation to sBP at baseline is presented in Figure 2(a). The absolute change in BP increased with increasing sBP at the start of the observation, ranging from a 15.7/9.3 (sBP/dBP) mmHg decrease at last visit in patients with sBP at baseline of ≥140 to <160 mmHg to a 46.3/17.9 (sBP/dBP) mmHg decrease at last visit in patients with a sBP at baseline of ≥180 mmHg.

The absolute changes in BP during the observation by age at baseline are shown in Figure 2(b). There were no major differences in BP decrease between the age groups.

In Table 4 the effects of therapy at the last visit compared with baseline (normalization of BP and response of BP) are summarized. A total of 43.5% of the patients reached normalization (BP <140/90 mmHg for non-diabetics or <130/80 mmHg for diabetics) and 71.3% showed a BP response (sBP <140 or ≥20 mmHg decrease vs baseline and dBP <90 or ≥10 mmHg decrease vs baseline in non-diabetic patients and sBP <130 or ≥20 mmHg decrease vs baseline and dBP <80 or ≥10 mmHg decrease vs baseline in patients with diabetes).

**Table 4.** Normalization of blood pressure and response at last visit.

	Total		Normalization*		Response**	
	*n*	%	*n*	%	*n*	%
Total	7108	100.0	3090	43.5	5067	71.3
Systolic blood pressure at start of observation						
<140 mmHg	661	100.0	405	61.3	413	62.5
≥140 and <160 mmHg	2711	100.0	1388	51.2	1626	60.0
≥160 and <180 mmHg	2660	100.0	992	37.3	2097	78.8
≥180 mmHg	1065	100.0	302	28.4	928	87.1
Diastolic blood pressure at start of observation						
<90 mmHg	2296	100.0	1094	47.6	1348	58.7
≥90 mmHg	4803	100.0	1993	41.5	3716	77.4
Age at start of observation						
<65 years	3319	100.0	1576	47.5	2475	74.6
≥65 years	3756	100.0	1497	39.9	2566	68.3
<80 years	6421	100.0	2831	44.1	4601	71.7
≥80 years	654	100.0	242	37.0	440	67.3

mmHg, millimeters of mercury; *n*, number. *Normalization = blood pressure < 140/90 mmHg for non-diabetics or <130/80 mmHg for diabetics. **Response = systolic blood pressure <140 mmHg or ≥20 mmHg decrease vs baseline and diastolic blood pressure <90 mmHg or ≥10 mmHg decrease vs baseline in non-diabetic patients and systolic blood pressure <130 mmHg or ≥20 mmHg decrease vs baseline and diastolic blood pressure <80 mmHg or ≥10 mmHg decrease vs baseline in patients with diabetes.

Normalization rates tended to decrease with increasing sBP at baseline, ranging from 61.3% in patients with sBP <140 mmHg to 28.4% in patients with sBP ≥180 mmHg.

In contrast, response rates tended to increase with increasing sBP at baseline (range = 60.0% in patients with sBP ≥140 to <160 mmHg to 87.1% in patients with sBP >180 mmHg). Similar tendencies were observed for stratification by dBP.

Normalization and response were roughly comparable in patients aged <65 years at baseline (normalization = 47.5%; response = 74.6%) compared with patients ≥65 years (normalization = 39.9%; response = 68.3%) and in patients <80 years (normalization =44.1%; response = 71.7%) compared with patients ≥80 years (normalization = 37.0%; response = 67.3%).

### Assessment of safety

Premature discontinuation of treatment occurred in 3.2% of the cases. The main reasons for these were adverse events (AEs, 42.9%) and change of compound (26.5%).

For 164 of the 7132 patients (2.3%) a total of 267 AEs (210 non-serious (nsAE) and 57 serious (SAE) adverse events) were recorded. Table 5 lists the most frequent AEs (i.e., ≥0.05% of patients). Peripheral edema (0.6%) and dizziness (0.2%) were the most common AEs. SAEs were only seen in 20 patients (0.3%), with most of these being individual occurrences.

**Table 5.** Most frequently reported adverse events (≥0.05% of patients).

	Total (*n* = 7132)	
	*n*	%
Edema peripheral	46	0.64
Dizziness	15	0.21
Edema	11	0.15
Nausea	10	0.14
Headache	10	0.14
Pruritus	5	0.07
Rash	4	0.06

*n*, number.

Three patients died during the observation period (myocardial infarction; multi-organ failure; pancreatic carcinoma). According to the treating physicians there was no causal relationship to the study drug in these cases.

## Discussion

The present study investigated the effectiveness and safety profile of A + V + H given as a single pill for the treatment of essential hypertension in daily practice. The efficacy of A + V + H combination therapy in hypertension treatment has been demonstrated in clinical trials^16,19–22^.

At baseline >90% of the patients included in this study had a sBP ≥140 mmHg and/or dBP ≥90 mmHg. In sBP the most commonly seen BP categories were ≥140 and <160 mmHg and ≥160 and <180 mmHg. The most frequent dBP category was ≥90 and <100 mmHg. The majority of patients were overweight or obese. Additionally, nearly all of the patients had CV risk factors, the most common being positive family history, dyslipidemia, and diabetes mellitus.

About two thirds of the patients in this study received A + V + H either in the combination of 5/160/12.5 mg or 10/160/12.5 mg. The dose was mostly stable throughout the observation period. If uptitration occurred, it was most often observed in increasing the A or V dosage. However, there is no information about the decision-making process.

During the observation period both sBP and dBP markedly decreased. Normalization (BP <140/90 mmHg or <130/80 mmHg for diabetics) was seen in ∼40% of the patients and ∼70% showed therapeutic response (sBP <140 or ≥20 mmHg decrease vs baseline and dBP <90 or ≥10 mmHg decrease vs baseline in non-diabetic patients and sBP <130 or ≥20 mmHg decrease vs baseline and dBP <80 or at least 10 mmHg decrease vs baseline in patients with diabetes). Taken together, these data show A + V + H is an effective drug in anti-hypertensive therapy in the patients studied.

The present study was an observational study investigating an heterogeneous patient population. Therefore, the average reduction in BP was in line with the expected effectiveness of A + V + H under real life conditions.

BP reduction was strongly dependent on the BP category at baseline. The highest decrease in BP (46.3/17.9 [sBP/dBP] mmHg) was seen in patients with a sBP of ≥180 mmHg at baseline, while patients with a sBP of ≥140 to <160 mmHg still had a decrease of 15.7/9.3 (sBP/dBP) mmHg. This effect is well known from clinical studies where patients with higher BP levels show a stronger response to initiation or intensification of anti-hypertensive therapy.

When analyzed by BP at the start of observation, the normalization rate at the last visit was higher in patients with lower BP compared with those with higher BP at the start of observation. In contrast, patients with elevated BP at baseline reached the therapeutic response more frequently at the last visit, most probably due to the fact that it was easier for them to fulfill the condition of ‘≥20 mmHg systolic and ≥10 mmHg diastolic BP reduction’ that was part of the definition of therapeutic response.

There was no major difference in BP decrease between the sub-groups by age, indicating comparable effectiveness of A + V + H across the age range of the study population. Similarly, normalization and response were roughly comparable for different age groups.

Most of the adverse events were observed in single cases only. In total, peripheral edema, which is a known side-effect of A, occurred most frequently. According to Fogari *et al*.^15^, the addition of V lowers the frequency of its occurrence. Taken together, treatment with A + V + H was safe and well tolerated.

The present study was an observational study and, therefore, has the inherent limitations and advantages associated with this type of study^23–25^.

First of all, lack of blinding and randomization may lead to a selection bias. The treating physician decides on the prescription and dosage of the respective medication, thereby influencing the patient and treatment groups. Second, the heterogeneity of the patient population may confound analysis due to a high variation in collected demographic and disease history data. Third, the lack of a control group complicates analysis of outcome data, especially the interpretation of BP response and adverse event rates. Furthermore, incomplete and inconsistent data due to patients that discontinue therapy or are lost to follow-up during the observation period may create an outcome bias. To minimize bias, the data of the last available visit after the start of observation were summed up as a last follow-up (i.e., called ‘last visit’).

Changes in laboratory parameters were not collected (e.g., liver and kidney tests) due to the non-interventional nature of this study. Availability of laboratory data would have allowed a more detailed assessment of safety and tolerability.

The calculation of normalization and therapeutic response used in this study was based on the hypertension guidelines in place at the time of the study^8^. The re-appraisal of these guidelines showed that the recommendation to lower BP <130/80 mmHg in patients with diabetes is not sufficiently supported by outcome trial evidence^9^. Thus, post-hoc analyses were performed to account for these new guidelines. According to these, normalization was defined as sBP <140/90 mmHg both in diabetic and non-diabetic patients and therapeutic response as sBP <140 or ≥20 mmHg decrease vs baseline and dBP <90 or ≥10 mmHg decrease vs baseline both in diabetic and non-diabetic patients. Normalization (BP <140/90 mmHg both in diabetic and non-diabetic patients) at last visit was seen in 58.6% of the patients and 79.7% showed therapeutic response (sBP <140 or ≥20 mmHg decrease vs baseline and dBP <90 or ≥10 mmHg decrease vs baseline both in diabetic and non-diabetic patients). As expected, normalization and response were higher according to the new guidelines.

Regarding normalization and response rates it has to be taken into account that 5.4% of the patients had already reached the target BP at baseline. It can be assumed that these patients were switching from the free combination therapy to fixed combination therapy.

However, a major strength of the study is the real life setting. Treatment decisions and selection of patients were performed by the physician according to practice routine and the Summary of Product Characteristics. Consequently, the results of the present study have a high generalizability and applicability. Furthermore, the collection of data in a real-life situation provides information on general data like typical patient characteristics and current treatment approaches in the management of hypertension in daily medical practice. Additionally, the sample size in this study was considered sufficiently large to allow analysis of outcome and differences between sub-groups.

This study showed that in a real world setting some patients still do not reach BP control. The physicians were not limited in treatment options, so that BP control theoretically could have been reached in a higher percentage of patients. Perhaps individual BP targets, which are often discussed and differ from the ones given in the official guidelines, are one of the reasons. Lack of individual patient compliance and ‘white coat hypertension’ could represent additional reasons.

In conclusion, the A + V + H single-pill combination effectively lowered BP in patients with essential hypertension and was well tolerated.

## Transparency

### Declaration of financial/other relationships

Andreas Hagendorff has received funding from GE Healthcare and Novartis and lecture fees from GE Healthcare, Philips, Siemens, Toshiba, Servier, and Novartis. Ira Kurz is employed by Kantar Health GmbH, which conducted the study and the analysis on behalf of Novartis Pharma GmbH. Sven Klebs is an employee of Novartis Pharma GmbH. Alfons Müller is an employee of Novartis Pharma GmbH.
